# PCV2 replication promoted by oxidative stress is dependent on the regulation of autophagy on apoptosis

**DOI:** 10.1186/s13567-019-0637-z

**Published:** 2019-03-05

**Authors:** Nianhui Zhai, Kai Liu, Hu Li, Zixuan Liu, Hong Wang, Viktor I. Korolchuk, Bernadette Carroll, Cuiling Pan, Fang Gan, Kehe Huang, Xingxiang Chen

**Affiliations:** 1 College of Veterinary Medicine, Nanjing Agricultural University, Nanjing, 210095 China; 2 Institute for Cell and Molecular Biosciences, Newcastle University, Newcastle upon Tyne, NE4 5PL UK; 3 Present Address: School of Biochemistry, University of Bristol, Bristol, BS8 1TS UK

## Abstract

Porcine circovirus type 2 (PCV2) is an economically important swine pathogen but some extra trigger factors are required for the development of PCV2-associated diseases. By evaluating cap protein expression, viral DNA copies and the number of infected cells, the present study further confirmed that oxidative stress can promote PCV2 replication. The results showed that oxidative stress induced autophagy in PCV2-infected PK15 cells. Blocking autophagy with inhibitor 3-methyladenine or ATG5-specific siRNA significantly inhibited oxidative stress-promoted PCV2 replication. Importantly, autophagy inhibition significantly increased apoptosis in oxidative stress-treated PK15 cells. Suppression of apoptosis by benzyloxycarbonyl-Val-Ala-Asp fluoromethylketone in conditions of autophagy inhibition restored PCV2 replication. Taken together, autophagy protected host cells against potential apoptosis and then contributed to PCV2 replication promotion caused by oxidative stress. Our findings can partly explain the pathogenic mechanism of PCV2 related to the oxidative stress-induced autophagy.

## Introduction

Porcine circovirus type 2 (PCV2), the smallest autonomously replicating animal virus, belongs to genus Circovirus of the *Circoviridae* family, and this virus is icosahedral, non-enveloped, with a covalently closed circular single-stranded DNA [1]. PCV2 is the primary causative agent of several syndromes known as PCV2-associated diseases [2], which has caused considerable economic loss in the swine industry [3]. However, not all pigs infected with PCV2 will develop PCV2-associated diseases. Actually, PCV2 alone rarely causes disease [4]. Several studies have reported that other trigger factors such as oxidative stress [5, 6], immune stimulation [7], presence of concurrent viral infections [8], mycotoxin [9, 10] and nutrition [11] could aggravate the infection but the related pathogenic mechanisms are still unclear.

Autophagy is an evolutionarily conserved catabolic process involved in the degradation and recycling of cytoplasmic components. It plays an essential role in normal development and responds to changing environmental stimuli [12, 13]. Generally, autophagy is considered to be a defense mechanism against some viral infection by removing intracellular pathogens [14]. Conversely, a number of viruses have evolved diverse strategies to subvert autophagy for their own replication [15, 16]. Some studies have shown that PCV2 infection triggers the autophagy pathway in host cells, which is essential for their own replication [17, 18]. Our previous studies demonstrated that oxidative stress can induce autophagy which facilitates PCV2 replication [6]. However, the mechanism involved in the promotion of PCV2 replication by oxidative stress-induced autophagy remains to be elucidated.

Apoptosis, also known as a programmed way of cell death, is an autonomous cell death based on a genetic program [13]. As a protective mechanism for the host, apoptosis plays an important role in maintaining the stability of the intracellular environment, regulating the differentiation of tissues and organs, and defending the cell against the infection with pathogenic microorganisms [19]. In some situation, the host cell can trigger apoptosis, a suicide way to protect the organism against the virus replication [20]. Inhibiting cellular apoptosis will facilitate some virus replication, assembly and spreading [21, 22]. Although autophagy and apoptosis are two completely different cell processes, previous studies suggested that autophagy and apoptosis interact with each other under certain conditions, and this dynamic balance may affect virus replication [23]. For instance, classical swine fever virus-induced autophagy delays apoptosis and thus contributes to the persistent viral infection in host cells [24]. However, it is still unknown whether autophagy interacts with apoptosis in the promotion of PCV2 replication induced by oxidative stress.

The aim of this study was to investigate the role of autophagy and apoptosis in oxidative stress-promoted PCV2 replication in PK15 cells.

## Materials and methods

### Reagents and antibodies

Bicinchoninic acid (BCA) protein assay kit (P0009), LDH cytotoxicity assay kit (C0016), enhanced chemiluminescence (ECL) kit (P0018M), MTT cell proliferation and cytotoxicity assay kit (C0009), Hoechst staining kit (C0003), benzyloxycarbonyl-Val-Ala-Asp fluoromethylketone (Z-VAD) (C1202), were obtained from Beyotime Institute of Biotechnology (Haimen, Jiangsu, China). Glutathione (GSH) assay kit was obtained from Nanjing Jiancheng Bioengineering Institute (Jiancheng, Nanjing, Jiangsu, China). Hydrogen peroxide (H_2_O_2_) and 3-methyladenine (3-MA) were obtained from Sigma-Aldrich (St. Louis, USA). Rabbit monoclonal anti-caspase-3 (cleaved) antibody was obtained from Beyotime Institute of Biotechnology. Rabbit polyclonal anti-LC3B antibody and horseradish peroxidase (HRP)-conjugated goat anti-rabbit or -mouse secondary antibodies were purchased from Sigma-Aldrich. Mouse monoclonal anti-β-actin antibody and rabbit polyclonal anti-ATG5 antibody were purchased from Santa Cruz Biotechnology (Santa Cruz, USA). X-tremeGENE siRNA transfection regent was from Roche (Basel, Switzerland).

### Cells culture and viruses propagation

Porcine kidney 15 (PK15) cells were gained from the China Institute of Veterinary Drug Control. PK15 cells were cultured in Dulbecco’s modified Eagle’s medium (DMEM, Invitrogen, Carlsbad, USA) supplemented with 8% newborn calf serum (NBCS), 100 μg/mL of streptomycin and 100 U/mL of penicillin at 37 °C with 5% CO_2_ in a humidified atmosphere.

The PCV2 strain (PCV2NJ2002) was isolated from a kidney tissue sample of a pig with naturally occurring postweaning multisystemic wasting syndrome (PMWS). The identification of PCV type was performed by sequencing (Invitrogen, USA). PCV2 was propagated in PK15 cells as previously described [6]. The virus was stored at −80 °C.

### MTT assay

PK15 cells were seeded in a 96-well plate at a density of 5 × 10^3^ cells/well for 24 h before the cells were treated with different concentrations of H_2_O_2_ for another 48 h. Cytotoxicity was determined using colorimetric MTT assay kit following the manufacturer’s protocol. Cells were treated with 3-(4, 5-cimethylthiazol-2-yl)-2, 5-diphenyl tetrazolium bromide (MTT) solution for 3 h. Then medium was replaced by 150 μL DMSO for 10 min. Optical density (OD) was measured by a spectrophotometer at 490 nm.

### LDH activity assay

PK15 cells were seeded in a 96-well plate at a density of 5 × 10^3^ cells/well for 24 h before the cells were treated with different concentrations of H_2_O_2_ for another 48 h. LDH activity was determined by LDH cytotoxicity assay kit. After treatment, the 96-well plate was centrifuged at 400 *g* for 5 min, and then the supernatant was collected and assayed following the manufacturer’s instructions. Optical density (OD) was measured by a spectrophotometer at 490 nm. LDH activity was normalized to protein concentrations and the results are expressed as percentage of the control values.

### Small interfering RNA (siRNA) transfection

ATG5-specific siRNA and Control siRNA sequences were designed by Invitrogen. The ATG5-specific siRNA sequence was 5′-GCUUCGAGAUGUGUGGUUUtt-3′, control siRNA sequence was 5′-CGUGUCACGUtt-3′. PK15 cells were transfected with the targeted siRNA using the X-treame GENE siRNA transfection agent (Roche) according to the protocol provided by the manufacturer. Transfection reagent was incubated for 5 h. The cells were then washed with DMEM and cultured for further treatments.

### Quantitative real-time PCR

PCV2 DNA copies in PK15 cells were determined by quantitative real-time PCR. After treatment, the TaKaRa DNA Mini kit (TaKaRa, Dalian, China) was used to extract the PCV2 DNA. The purified DNA was used as a template for real-time PCR amplification. A 117-bp region was amplified from PCV2 gene with a pair of PCV2-specific primers (forward primer: 5′-TAGTATTCAAAGGGCACAG-3′, reverse primer: 5′-AAGGCTACCACAGTCAG-3′). Quantitative real-time PCR was performed using the ABI Prism Step One Plus detection system (Applied Biosystems, Foster city, USA). A recombinant pMD19 plasmid vector (TaKaRa) containing a PCV2 genome insert as a standard reference and PCV2 viral DNA was measured by the TaKaRa SYBR green real-time PCR kit.

### Cytokine mRNA Levels by Real-Time PCR

The primers used were synthesized by Invitrogen (Paisley, Scotland, UK) (Table 1). PK15 cells were plated in a 12-well plate at a density of 5 × 10^4^ cells/well. After treatment, total RNA was isolated using the RNAiso Plus kit (TaKaRa) according to the manufacturer’s instructions. The RNA quality was evaluated by the ratio of OD260/OD280. Expression of genes was evaluated by real-time polymerase chain reaction (PCR) using the ABI Prism Step One Plus detection system (Applied Biosystems). A no-template control served as the negative control. The relative mRNA levels of each cytokine were assessed using the 2^−ΔΔCT^ method and normalized to the housekeeping gene β-actin. Real time PCR was performed using SYBR Premix Ex TaqII (TaKaRa) and the ABI 7500 realtime PCR system.

**Table 1:** **Primers used in this study**

Gene	Primer sequence (5′-3′)
β-actin	F: CTGCGGCATCCACGAAACT
	R: AGGGCCGTGATCTCCTTCTG
Caspase-3	F: GGAATGGCATGTCGATCTGGT
	R: ACTGTCCGTCTCAATCCCAC
Caspase-8	F: TCTGCGGACTGGATGTGATT
	R: TCTGAGGTTGCTGGTCACAC
Bax	F: ATGATCGCAGCCGTGGACACG
	R: ACGAAGATGGTCACCGTCGC
Bcl-2	F: GAAACCCCTAGTGCCATCAA
	R: GGGACGTCAGGTCACTGAAT

### Indirect immunofluorescence assay (IFA)

PCV2-infected PK15 cells were assayed by IFA. PK15 cells were plated in a 96-well plate at a density of 5 × 10^3^ cells/well. After treatment, PK15 cells were fixed in 4% paraformaldehyde for 20 min. After washed with PBST three times, the cells were permeabilised with 0.1% Triton X-100 for 20 min and incubated in PBST containing 1% bovine serum albumin (BSA) at 37 °C for 1 h to prevent nonspecific binding. Then, the cells were incubated with porcine anti-PCV2 antibody (UnivBiotech, Shanghai, China) at 37 °C for 1 h, and after three times washes with PBST, FITC-conjugated rabbit anti-pig antibody (Sigma) was added and incubated at 37 °C for 1 h. After three washes, the cells were observed under a fluorescence microscope. Cells positive for PCV2 viral antigens were counted in six fields of view. The calculation of the relative proportions of infected cells was based on the total amount of cells in each field.

### Determination of glutathione and intracellular ROS

To detect the production of GSH, cells were harvested with PBS and then sonicated (Sonics VCX105). The cell homogenate was centrifuged at 12 000 rpm at 4 °C for 15 min. The supernatant was collected, and the absorbance was assessed at 405 nm by glutathione (GSH) assay kit (Jiancheng, Nanjing, Jiangsu, China).

The amount of intracellular reactive oxygen species (ROS) was measured by the reactive oxygen species assay kit (Beyotime) according to the manufacturer instructions. Cells were incubated with DCFH-DA probes (10 μM) for 30 min at 37 °C in the dark. The ROS level was measured by fluorescence microscopy at 488 nm (excitation) and 581 nm (emission), and analyzed by Image-Pro Plus 6.0 software (Media Cybernetics, Sarasota, USA).

### Western blotting analysis

The expression level of cleaved caspase-3, ATG5, LC3, cap, and β-actin were examined by Western blot. Cells were incubated under control and experimental conditions. After treatment, cells were collected and washed three times with ice-cold PBS followed by resuspension in RIPA buffer to prepare the whole cell lysates followed by centrifugation at 12 000 rpm for 20 min at 4 °C. Protein concentrations were determined by the BCA protein assay kit (Beyotime Jiangsu, China). A total of 30 μg of protein samples were diluted in 5× SDS-PAGE loading buffer and heated at 95 °C for 5 min. The samples were separated on 12% SDS-PAGE gels and transferred onto polyvinylidene fluoride (PVDF) membranes (Millipore, Molsheim, France). After blocking with 5% (w/v) BSA in TBST for 2 h, the membranes were incubated with primary antibodies overnight at 4 °C. The membranes were washed with TBST three times and incubated with secondary antibody for 1 h at room temperature. Blots were visualized according to the standard enhanced chemiluminescence system (Bio-Rad, Berkeley, USA). Quantification of protein blots was performed using the Image-Pro Plus 6.0 software (Media Cybernetics, Sarasota, USA), and images were acquired from an EU-88 image scanner (Seiko Epson Corporation, Suwa, Japan).

### Analysis of apoptosis by Hoechst 33258 staining

Apoptotic was detected by staining with the DNA binding fluorochrome Hoechst 33258. After treatment, cells were fixed with 4% formaldehyde. After staining with Hoechst solution, cells were washed again and observed under a fluorescence microscope (Zeiss) at 340 nm.

### Statistical analysis

Statistical analyses were performed using the GraphPad Prism version 6.0 (GraphPad Software, San Diego, CA, USA). All experiments were repeated at least three times and each experiment was carried out at least by triplicates. Data were analyzed using one-way analysis of variance (ANOVA) followed by least-significant difference test. Data are expressed as the mean ± SEM. *P*  <  0.05 were considered statistical significant.

## Results

### Cytotoxic effects of H_2_O_2_ on PK15 cells

**Figure 1:**
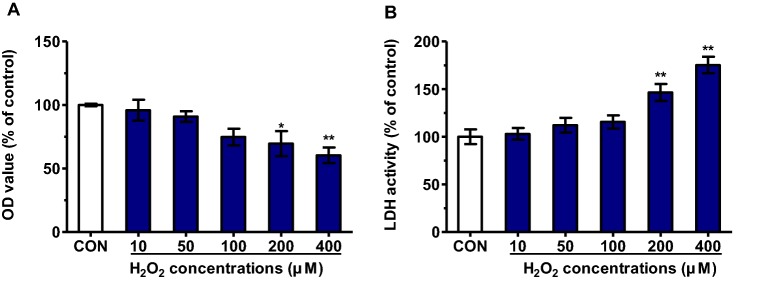
**Cytotoxic effects of H**_**2**_**O**_**2**_ **on PK15 cells.** PK15 cells were plated in 96-well plates at a density of 5 × 10^3^ cells/well and cultured with H_2_O_2_ at different concentrations for 48 h. Cell viability were determined by MTT assay (**A**) and LDH activity assay (**B**). All results are presented as mean ± SEM (*n* = 5, each). Significance compared with control (*P < 0.05 and **P < 0.01).

The toxicity of H_2_O_2_ was assessed by MTT assay and LDH assay. PK15 cells were incubated with increasing concentrations of H_2_O_2_ (0–400 µM) for 48 h. As shown in Figure 1A, after H_2_O_2_ exposure, there is a dose-dependent decrease in cell viability. The amounts of LDH in the supernatant medium indirectly reflect H_2_O_2_-induced cytotoxicity. As shown in Figure 1B, the release of LDH was significantly increased after cells were treated with H_2_O_2_ at the concentrations of 200 µM and 400 µM, compared to the control group (*P* < 0.01). Therefore, H_2_O_2_ was used at the concentrations of 10, 50 and 100 µM for subsequent experiments.

### Oxidative stress promotes PCV2 replication and induces autophagy in PCV2-infected cells

**Figure 2:**
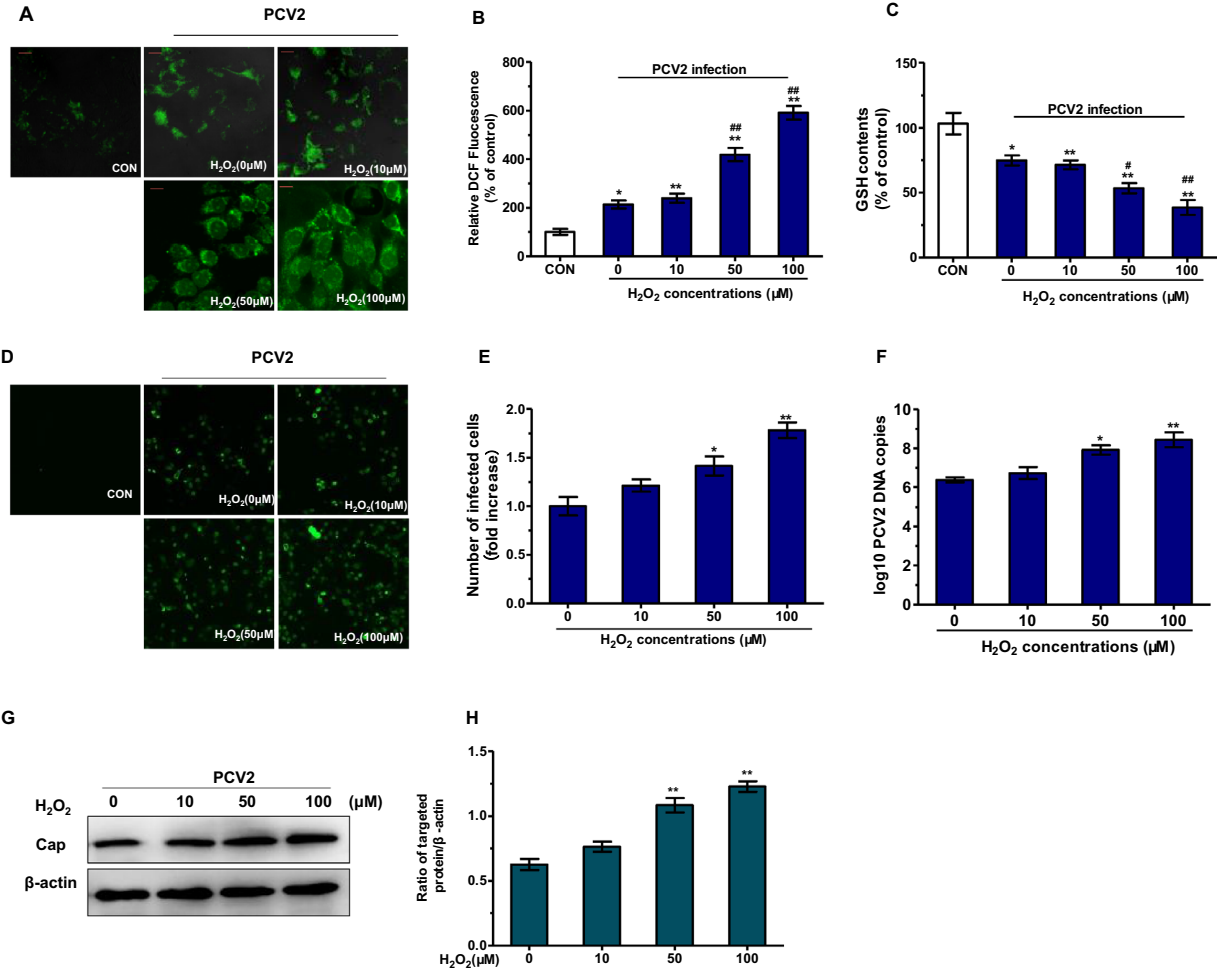
**Oxidative stress promotes PCV2 replication in PCV2-infeced PK15 cells.** PK15 cells were infected with PCV2 at MOI of 1, after 2 h infection, the cells were further cultured in fresh medium with different concentrations of H_2_O_2_ for 48 h. **A**, **B** Cells were stained with H2DCF-DA to measure intracellular ROS. **C** The determination of GSH content was performed according to the procedures described in materials and methods. **D**, **E** The number of infected cells was detected by immunofluorescence assay. **F** Real-time PCR was used to detect the PCV2 viral DNA copies. **G**, **H** The expression of viral cap protein was detected by Western blotting. Scale bar: 10 µm. All results are presented as mean ± SEM (*n* = 3, each). Significance compared with control (*P < 0.05 and **P < 0.01). Significance compared with single PCV2-infected group (^#^*P* < 0.05 and ^##^*P* < 0.01).

To investigate the effects of oxidative stress on PCV2 replication, H_2_O_2_ was used to induce oxidative stress in PK15 cells. Cells were inoculated with PCV2 at a multiplicity of infection (MOI) of 1 and incubated with H_2_O_2_ for 72 h. As shown in Figures 2A–C, 50 µM and 100 µM H_2_O_2_ treatments induced a significant increase in ROS level and a significant decrease in intracellular GSH level compared to the single PCV2-infected group (*P* < 0.01). Furthermore, H_2_O_2_ treatment promoted PCV2 replication. 50 µM and 100 µM H_2_O_2_ significantly increased the number of PCV2-infected cells, the PCV2 DNA copies and the viral cap protein level compared to the single PCV2-infected group (Figures 2D–H) (*P* < 0.01). These results indicate that H_2_O_2_-induced oxidative stress promotes PCV2 replication.

**Figure 3:**
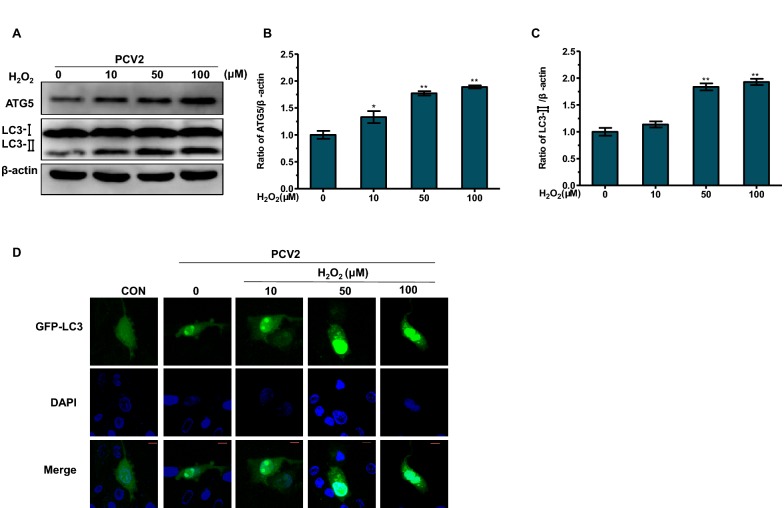
**Oxidative stress induces autophagy in PCV2-infected PK 15 cells.** PK15 cells were infected with PCV2 at MOI of 1, after 2 h infection, the cells were further cultured in fresh medium with different concentrations of H_2_O_2_ for 48 h. **A**–**C** The expression of ATG5 and LC3-II protein was detected by Western blotting. **D** Cells were transfected with GFP-LC3 plasmid and analyzed by fluorescence microscopy for the presence of fluorescent GFP-LC3 puncta. Scale bar: 10 μm. All results are presented as mean ± SEM (*n* = 3, each). Significance compared with single PCV2-infected group (*P < 0.05 and **P < 0.01).

Then we determined whether oxidative stress treatment in PCV2-infected PK15 cells can induce autophagy. Western blot assay was used to measure the conversion of LC3 and the expression of protein ATG5 since they are the autophagy markers. As shown in Figures 3A–C, H_2_O_2_ treatment significantly increased the expression of protein LC3-II and ATG5. Then PK15 cells were transfected with green fluorescent protein-microtubule associated protein 1 light-chain 3 (GFP-LC3), a specific marker of autophagic vesicles and autophagic activity. The results showed that H_2_O_2_ treatment upregulated the accumulation of the GFP-LC3 dots, which was consistent with the result of Western blot assay. These results show that oxidative stress could promote PCV2 replication and induce autophagy in PCV2-infected cells.

### Autophagy inhibition decreases oxidative stress-promoted PCV2 replication

**Figure 4:**
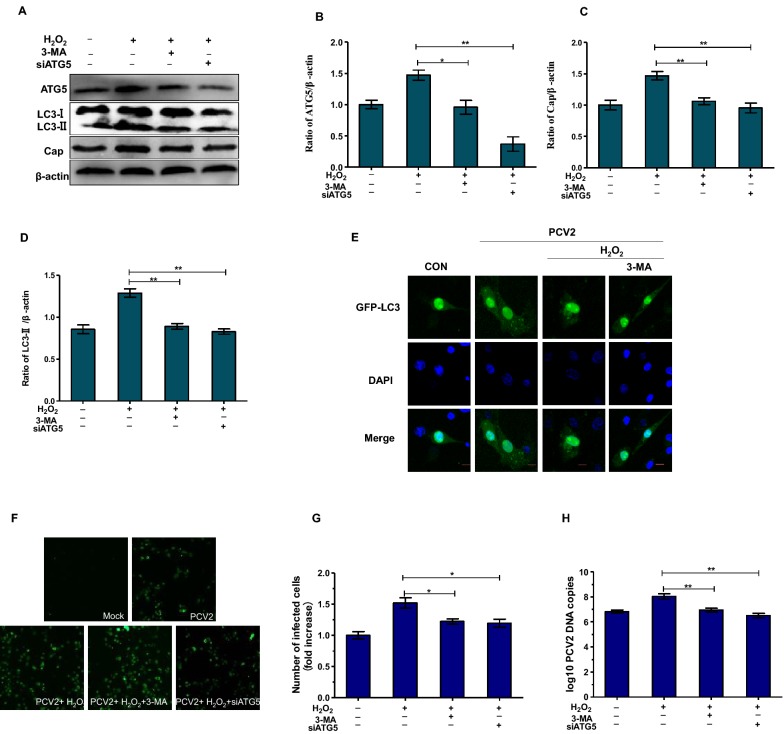
**Autophagy inhibition decreases oxidative stress-promoted PCV2 replication.** PK15 cells were infected with PCV2 at MOI of 1, cells were pretreated with 3-MA (5 mM) or transfected with the siATG5 and then cultured with different concentrations of H_2_O_2_ for 48 h. **A**–**D** The expression of ATG5 and LC3-II protein was detected by Western blotting. **E** Cells were transfected with GFP-LC3 plasmid and analyzed by fluorescence microscopy for the presence of fluorescent GFP-LC3 puncta. Scale bar: 10 μm. **F**, **G** The number of infected cells was detected by immunofluorescence assay. **H** Real-time PCR was used to detect the PCV2 viral DNA copies. All results are presented as mean ± SEM (*n* = 3, each). Significance compared with PCV2-infected and H_2_O_2_ treatment group (*P < 0.05 and **P < 0.01).

To confirm the role of autophagy mechanism in oxidative stress-promoted PCV2 replication, autophagy inhibitor 3-MA or small interfering RNAs (siRNAs) targeting ATG5 were used to block autophagy. As demonstrated in Figures 4A–D, Both the 3-MA treatment and ATG5 knockdown significantly reduced the ATG5 and LC3-II protein level compared to H_2_O_2_ treatment group (*P* < 0.01), and 3-MA treatment also downregulated the accumulation of the GFP-LC3 dots. Furthermore, 3-MA treatment and ATG5 knockdown significantly reduced viral cap protein level, the PCV2 DNA copies and the number of PCV2-infected cells, compared to the single PCV2-infected group (*P* < 0.05). These results show that autophagy inhibition could block oxidative stress-promoted PCV2 replication.

### Autophagy inhibition increases apoptosis in PCV2-infected PK15 cells

**Figure 5:**
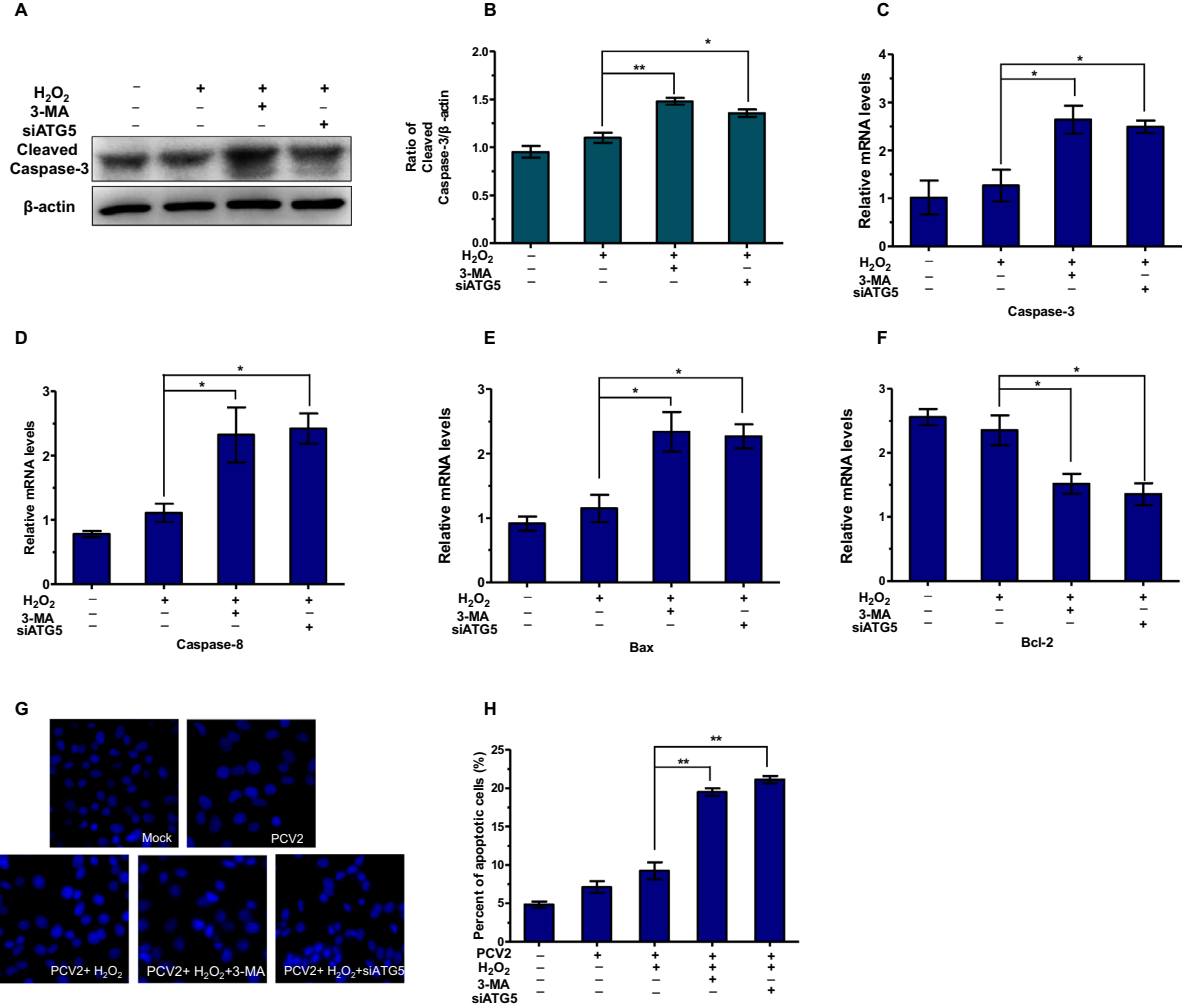
**Autophagy inhibition increases apoptosis in PCV2-infected PK15 cells.** PK15 cells were infected with PCV2 at MOI of 1, cells were pretreated with 3-MA or transfected with the siATG5 and then cultured in the presence of H_2_O_2_ for 48 h. **A**, **B** The expression of cleaved caspase-3 protein was detected by Western blotting. **C**–**F** The mRNA levels of caspase-3, caspase-8, Bax and Bcl-2 was measured by RT-PCR. **G**, **H** Morphology of apoptotic cell nuclei was detected by staining Hoechst 33258. The data represent the mean ± SEM (*n* = 3, each). Significance compared with PCV2-infected and H_2_O_2_ treatment group (*P < 0.05 and **P < 0.01).

Increasing evidence suggests that in some instances autophagy and apoptosis are cross inhibitory. To determine how oxidative stress-induced autophagy affects the infection of PCV2 in PK15 cells, apoptosis mechanism was investigated. We blocked autophagy with 3-MA or siATG5. Western bolt assay was used to detect the expression of apoptosis protein cleaved caspase-3. As shown in Figures 5A and B, autophagy inhibition significantly increased the expression of cleaved caspase-3 protein (*P* < 0.05). The mRNA levels of caspase 3, caspase 8, Bax and Bcl-2 were also detected by RT-PCR. Treatment with 3-MA or transfection with the siATG5 significantly increased the mRNA levels of caspase 3, caspase 8 and Bax, but decreased the Bcl-2 mRNA levels (*P* < 0.05).

The morphological changes of apoptotic cells were detected using Hoechst 33258 staining. Apoptosis was defined by morphologic changes in the nuclei such as chromatin condensation and nuclear fragmentation. As shown in Figure 5G, the number of cells with apoptotic characteristics enhanced when autophagy was suppressed. Similar results were obtained when autophagic activity was inhibited by knocking down ATG5. These results indicate that oxidative stress-induced autophagy could attenuate apoptosis.

### Inhibition of apoptosis restores oxidative stress-promoted PCV2 replication

**Figure 6:**
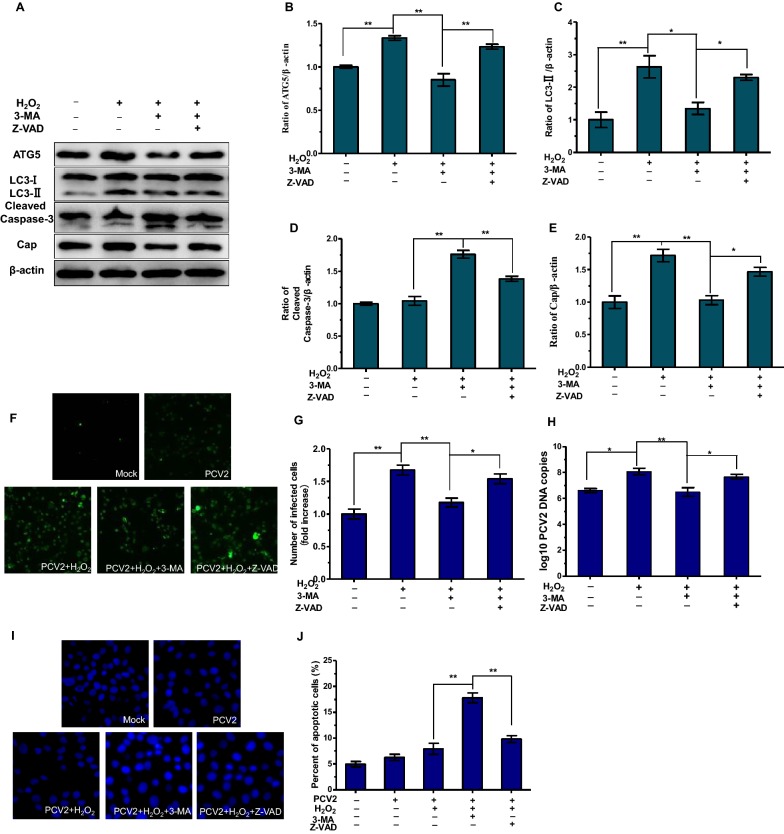
**Inhibition of apoptosis restores oxidative stress-promoted PCV2 replication.** PK15 cells were infected with PCV2 at MOI of 1, cells were pretreated with 3-MA or pretreated with 3-MA and Z-VAD (10 µM), then cultured with H_2_O_2_. **A**–**E** The expression of ATG5, LC3-II, cleaved caspase-3 and cap protein were detected by Western blotting. **F**, **G** The number of infected cells was detected by immunofluorescence assay. **H** Real-time PCR was used to detect the PCV2 viral DNA copies. **I**, **J** Morphology of apoptotic cell nuclei was detected by staining Hoechst 33258. The data represent the mean ± SEM (*n* = 3, each). Significance represent as *P < 0.05 and **P < 0.01.

To further confirm whether autophagic machinery influences apoptosis during PCV2 infection, Z-VAD, an inhibitor of apoptosis was used. As shown in Figures 6A–E, suppression of apoptosis by Z-VAD in conditions of autophagy inhibition rescued host cells. Notably, Z-VAD treatment partially reversed the effects of autophagy inhibition on PCV2 replication (*P* < 0.05) (Figures 6F–H). These results indicate that autophagy inhibition does not decrease persistent PCV2 infection if apoptosis is blocked.

## Discussion

PCV2 is an emerging viral disease and a major factor affecting pig health [25]. It is linked to PCV2-associated diseases, which includes PMWS [26], porcine dermatitis and nephropathy syndrome (PDNS) [27] and porcine respiratory disease complex (PRDC) [28]. The clinical signs of PCV2-associated diseases are nonspecific and variable. It can be subclinical or include one or more clinical manifestations [26]. Numerous cofactors have been shown to contribute to the development of PCV2-associated diseases. However, to date, the pathogenesis of PCV2 is still unclear.

Intracellular redox state is the result of mutual antagonism between the oxidant and the antioxidant processes [29]. Many studies have determined that ROS, key molecules accumulation of which leads to oxidative stress, can induce autophagy [30–32]. Both oxidative stress and autophagy may affect viral replication in the host cells [33]. It has been shown that PCV2 infection could cause ROS accumulation [6]. In addition, autophagy induced by oxidative stress and PCV2 infection itself can also promote PCV2 infection and replication [9, 10, 17, 34, 35]. In this study, oxidative stress has been further confirmed to promote PCV2 replication in PK15 cells (Figure 2). Oxidative stress caused autophagy in PK15 cells by detecting the expression of LC3-II, ATG5, and the accumulation of GFP-LC3 dots (Figure 3). In contrast, this effect was suppressed by inhibiting autophagy by 3-MA or transfection with the siATG5 (Figure 4). These results suggest that oxidative stress could induce autophagy in PCV2-infected PK15 cells which is responsible for PCV2 replication promotion.

Apoptosis and autophagy can antagonize or assist each other, thus differentially influencing cell fate. Many studies have shown the importance of the interaction between autophagy and apoptosis. For instance, Robin et al. have shown that differential expression of isoforms of pro-apoptotic protein TP53/p53, combined with the antagonism between apoptosis and autophagy, ensured the response to stress [36]. Beclin 1, an upstream molecule required for autophagosome formation, also regulates apoptosis by interacting with Bcl-2 [37]. Obviously, in some cases, the same proteins have been shown as a link between autophagy and apoptosis, which regulates both autophagic and apoptotic processes at the cellular level and these processes can interact with each other under different environmental conditions. Previous study has shown that chikungunya virus can delay caspase-dependent apoptosis by inducing autophagy [38], indicating that autophagy favors the survival of host cells through the regulation of apoptosis and thus contributes to virus propagation. Previous studies have also shown that respiratory syncytial virus can increase cellular ROS levels and induce autophagy, but autophagy inhibition significantly deceased cell viability and increased cell apoptosis in HEp-2 cells [39]. Our results showed that autophagy inhibition decreased PCV2 replication and induced apoptosis (Figures 4 and 5). Z-VAD, an apoptosis inhibitor, can partially reverse the effects of autophagy inhibition on PCV2 replication (Figure 6). These results indicate that oxidative stress-induced autophagy mediated the inhibition of apoptosis, which is beneficial for the continued survival of host cells and persistent replication of PCV2. The role of apoptosis in PCV2 infection has also been demonstrated in other study, in which PCV2 ORF4 protein was found to directly inhibit cell apoptosis and then facilitate the early infection of PCV2 [40].

In summary, this study reveals that autophagy induced by oxidative stress promotes PCV2 replication in PK15 cells through inhibiting the apoptosis pathway and therefore contributes to persistent viral infection. Our study provides a novel insight into the role of interplay between autophagy and apoptosis in PCV2 replication promoted by oxidative stress. Our findings can partly interpret the pathogenic mechanism of PCV2 related to the oxidative stress-induced autophagy.

## Abbreviations

PCV2: porcine circovirus type 2
ROS: reactive oxygen species
Z-VAD: benzyloxycarbonyl-Val-Ala-Asp fluoromethylketone
H_2_O_2_: hydrogen peroxide
NBCS: newborn calf serum
MTT: 3-(4, 5-dimethylthiazol-2-yl)-2, 5-diphenyl-2H-tetrazolium bromide
DMEM: Dulbecco’s modified Eagle’s medium
3-MA: 3-methyladenine
ANOVA: one-way analysis of variance
IFA: Indirect immunofluorescence assay
PBS: phosphate-buffered saline
PMWS: postweaning multisystemic wasting syndrome
MOI: multiplicity of infection
